# Hedgehog-PKA Signaling and gnrh3 Regulate the Development of Zebrafish gnrh3 Neurons

**DOI:** 10.1371/journal.pone.0095545

**Published:** 2014-05-30

**Authors:** Ming-Wei Kuo, Show-Wan Lou, Bon-chu Chung

**Affiliations:** 1 Institute of Molecular Biology, Academia Sinica, Taipei, Taiwan; 2 Institute of Fisheries Science, National Taiwan University, Taipei, Taiwan; Institute of Cellular and Organismic Biology, Taiwan

## Abstract

GnRH neurons secrete GnRH that controls the development of the reproduction system. Despite many studies, the signals controlling the development of GnRH neurons from its progenitors have not been fully established. To understand the development of GnRH neurons, we examined the development of *gnrh3*-expressing cells using a transgenic zebrafish line that expresses green fluorescent protein (GFP) and LacZ driven by the *gnrh3* promoter. GFP and LacZ expression recapitulated that of *gnrh3* in the olfactory region, olfactory bulb and telencephalon. Depletion of *gnrh3* by morpholinos led to a reduction of GFP- and gnrh3-expressing cells, while over-expression of *gnrh3* mRNA increased the number of these cells. This result indicates a positive feed-forward regulation of gnrh3 cells by gnrh3. The gnrh3 cells were absent in embryos that lack Hedgehog signaling, but their numbers were increased in embryos overexpressing *shhb*. We manipulated the amounts of kinase that antagonizes the Hedgehog signaling pathway, protein kinase A (PKA), by treating embryos with PKA activator forskolin or by injecting mRNAs encoding its constitutively active catalytic subunit (PKA*) and dominant negative regulatory subunit (*PKI*) into zebrafish embryos. PKA* misexpression or forskolin treatment decreased GFP cell numbers, while *PKI* misexpression led to ectopic production of GFP cells. Our data indicate that the Hedgehog-PKA pathway participates in the development of gnrh3-expressing neurons during embryogenesis.

## Introduction

GnRH is a neuropeptide that stimulates the secretion of gonadotropins from the pituitary; it controls reproduction via a neuroendocrine system. Three vertebrate *GnRH* genes are expressed at different brain regions [1]. GnRH1 (LHRH) is primarily found in the hypothalamus, GnRH2 (cGnRH-II) in the midbrain, while GnRH3 (sGnRH) mainly in the terminal nerve of telencephalon [2]. Zebrafish genome lacks *GnRH1*, while human genome lacks *GnRH3*. GnRH2 neurons are expressed in the midbrain from fish to mammals [2]. Deletion of *GnRH1* gene or disruption of the migration of GnRH1-expressing cells results in hypogonadal mouse with reproductive dysfunction [3], [4], showing the importance of GnRH1 in the development of reproductive organs. GnRH2 and GnRH3 cells appear to modulate sexual behaviors [5]–[7].

In vertebrates, while GnRH2 cells are originated locally in the midbrain, GnRH1 and GnRH3 neurons migrate from their origins to the final destinations in the hypothalamus and the terminal nerve, respectively [8]. The neuroendocrine cells originated from adenohypophysis form the future gnrh1 cells in the hypothalamus, while zebrafish neuromodulatory cells of the neural crest origin form the future gnrh3 cells in the terminal nerve [8]. GnRH3-producing cells migrate from the olfactory region via terminal nerve into the olfactory bulb and the preoptic area of the brain in salmon [9]–[11], medaka [12], barfin flounder [13], European sea bass [14] and zebrafish [15].

It is not clear how gnrh progenitor cells differentiate into GnRH neurons, except it is regulated by cues in the nasal midline [16]. In the mouse nasal placode, fibroblast growth factors (FGFs) stimulate the differentiation and axon targeting of GnRH neurons [17]. Blocking FGF signaling in GnRH neurons leads to reduced numbers of GnRH neurons, although the anatomical distribution of GnRH neurons was unaltered [18]. Other factors like retinoic acid, FGF8, Sonic hedgehog (SHH), bone morphogenetic proteins and transcription factor Lhx2 are also involved in the patterning and differentiation of the olfactory system [19], [20].

The Hedgehog (Hh) pathway has been implicated in the development of olfactory neurons. Loss of *Shh* disrupts the axon trajectory of the olfactory receptor neurons [21]. Loss of Xenopus *Xhip*, an Hh-specific antagonist in Xenopus, suppresses the formation of olfactory placode, while its overexpression results in a larger olfactory placode [22]. In this report we have examined the development of *gnrh3* neurons *in vivo* by analyzing a *gnrh3*-*GFP/LacZ* transgenic fish line that we generated. We showed that the development of zebrafish gnrh3 neurons was regulated by gnrh itself and by the Hedgehog-PKA pathway, and FGF signaling may also affect this process.

## Materials and Methods

### Animals

Zebrafish of the AB and TL strains were reared at 28.5°C as described [23]. The protocol for the use of zebrafish was approved by the Academia Sinica Institutional Animal Care and Utilization Committee. The transgene construct was injected into fish of the AB background to generate F0 transgenic fish, which was crossed with wildtype AB strain to generate F1 transgenic fish. These F1 fish were crossed with TL to obtain F2 transgenic fish in the AB/TL background. The F2 transgenic fish were intercrossed, and the homozygous F4 transgenic fish were examined for all experiments reported here. The *smu* ^b577^ (*slow muscle omitted*, Hh signaling component), *cyc* ^b16^ (*cyclops*, nodal related), and *oep* ^m134^ (*one-eyed pinhead*, nodal coreceptor) mutants have been previously described [24]–[26]. To prevent pigmentation, 0.2 mM phenylthiourea (PTU) was added to the water on day 1 post-fertilization (dpf). PTU treatment did not affect *gnrh3* gene expression as GFP and LacZ staining with or without PTU had the same pattern (Fig. S1 in File S1).

### In situ Hybridization and Immunofluorescence

Whole-mount in situ hybridization was performed using digoxigenin-labeled antisense RNA probes followed by detection with anti-digoxigenin alkaline phosphatase-conjugated antibody as described previously [27]. The *pGEM-T-gnrh3* plasmid was linearized with NcoI before being used as a template for *in vitro* transcription with SP6 RNA polymerase. After *in situ* hybridization, staining and mounting, images were captured with a digital camera (Coolpix 990, Nikon).

For double staining, after *in situ* hybridization, digoxigenin-labeled *gnrh3* antisense RNA probe was first reacted with mouse anti-Digoxin conjugated DyLight 488 (Jackson ImmunoResearch Laboratories, West Grove, PA, USA) followed by immunochemical reaction with rabbit anti-GFP antibody (sc-8334, Santa Cruz, CA, USA) and detection with Alexa Fluor 647-conjugated to donkey anti-rabbit IgG antibody (Invitrogen Corporation, Carlsbad, CA, USA). The signals were observed using a Leica TCS-SP5-MP-SMD confocal system (Leica Microsystems Wetzlar, Wetzlar, Germany).

### Gnrh3 Gene Cloning, Reporter Gene Constructs and Microinjection

Genomic clones containing the *gnrh3* gene were isolated from a zebrafish BAC genomic DNA library (Incyte Genomic, St. Louis, Missouri) using a *gnrh3* cDNA fragment as the probe. Four clones (81d01, 131o09, 135e16 and 148c10) were analyzed, and clones 135e16 and 148c10 were further subcloned. About a 10-kb region covering the entire *gnrh3* gene and its 5′- and 3′-flanking regions has been completely sequenced several times. The sequence is the same as that reported in GenBank (accession number AJ304429). To create a zebrafish *gnrh3-GFP* targeting vector, the 2.7-kb fragment containing the *gnrh3* promoter to the ATG and the 2.6-kb fragment containing ATG to *gnrh3* downstream were each ligated into the multiple cloning site of *pChi-GZK* vector (a gift from Dr. Guor-Mour Her) that contains the genes for LacZ and GFP. The final targeting construct, *pChi-gnrh3-GZK*, was about 15 kb (Fig. S2 in File S1). Transgenic zebrafish were generated by microinjection of 50–100 pg *pChi-gnrh3-GZK* DNA into embryos at the one- or two-cell stage. The reporter gene expression was monitored from 24 hpf to 10 dpf using a fluorescence dissecting microscope (MZ-FLIII with GFP 2 filter, Leica). Fluorescence images were captured with a cooling digital camera (SPOT, DIAGNOSTIC instrument, Inc.).

### Morpholino and mRNA Microinjections

Morpholinos of *gnrh3* antisense MO1 (5′-cactccatgctaaaactgctgtgtt-3′), MO2 (5′-ggaccagcaaccttcctttccactc-3′), MO3 (5′-gcaaccttcctttccactccatgct-3′), control MO1 (5′-aacacagcagttttagcatggagtg-3′), *csnk1a* (5′-ccatgtcctaaaatccgagaagtac-3′), *gsk3b* (5′- gagtaaaatacgtctgtttgtcttg-3′), control MO2 (5′-cctcttacctcagttacaatttata-3′) and *fgfr1* (5′-gcagcagcgtggtcttcattatcat-3′) (GeneTools, Corvallis, Oregon) were diluted to 10 ng/nl and injected into the yolk of 1-cell embryos at 3–15 ng/injection. Capped RNA was synthesized with mMESSAGE mMACHINE T7/SP6 kit (Ambion, Austin, Taxas) from linearized plasmids. Full-length *gnrh3* (100 pg) [1], *gnrh2* (100 pg) [1], constitutively active PKA catalytic subunit PKA* (50 pg) [28], *PKI* (dominant-negative regulatory subunit of *protein kinase A*, 100 pg) [28], *shha* (2 ng) [29] and *shhb* (*twhh*, 2 ng) [29] mRNA was injected into the yolk of 1-cell embryos, and embryos were allowed to develop at 28.5°C.

### β-*galactosidase* Histochemistry

For the detection of LacZ activity, larvae were fixed with 4% paraformaldehyde in PBS for 5–10 min at 4°C, washed with PBS, and then incubated in 1 mg/ml LacZ substrate, Bluo-gal (5-bromo-indolyl-b-O-galactopyranoside), in reaction buffer (3 mM potassium ferricyanide, 3 mM potassium ferrocyanide, 1.5 mM magnesium sulfate, 0.2% sodium deoxycholate, 0.1% Nonidet P-40, and 0.15 mg/ml chloroquine in PBS) at room temperature overnight or two days [30].

### Drug Treatment

Embryos were soaked in forskolin (200 µM, Sigma), cyclopamine (50 or 100 µM), tomatide (100 µM in EtOH, Calbiochem), or LiCl (200 µM) at 28.5°C from 6 hpf to 10 hpf or indicated otherwise.

### Statistical Analysis

The total number of GFP expressed as means±standard error of the mean (S.E.M.). *P<0.05, **P<0.01.

## Results

### Transgenic GFP and LacZ Expression can Trace gnrh3 Neurons

**Figure 1:**
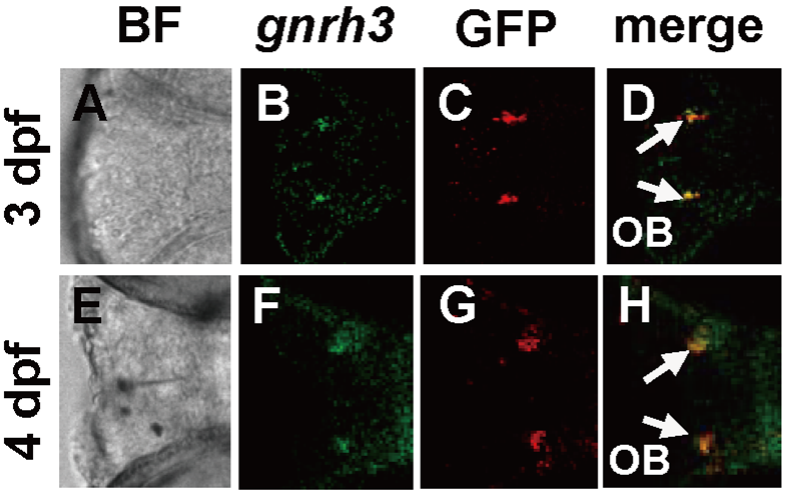
Co-localization *gnrh3* mRNA and GFP in transgenic fish expressing *GFP-LacZ* under the control of the *gnrh3* promoter. A–D, 3 dpf, E–H, 4 dpf. A and E, bright field (BF) view of the embryos. B and F, green color indicated *gnrh3* mRNA detection by *in situ* hybridization. C and G, GFP immunostaining is shown as red signal. D and H, the merged pictures show co-localization of *gnrh3* and GFP signals in the olfactory bulb (OB) at 3 dpf and 4 dpf. The anterior is to the left in all panels, and arrows point to the olfactory bulb.

**Figure 2:**
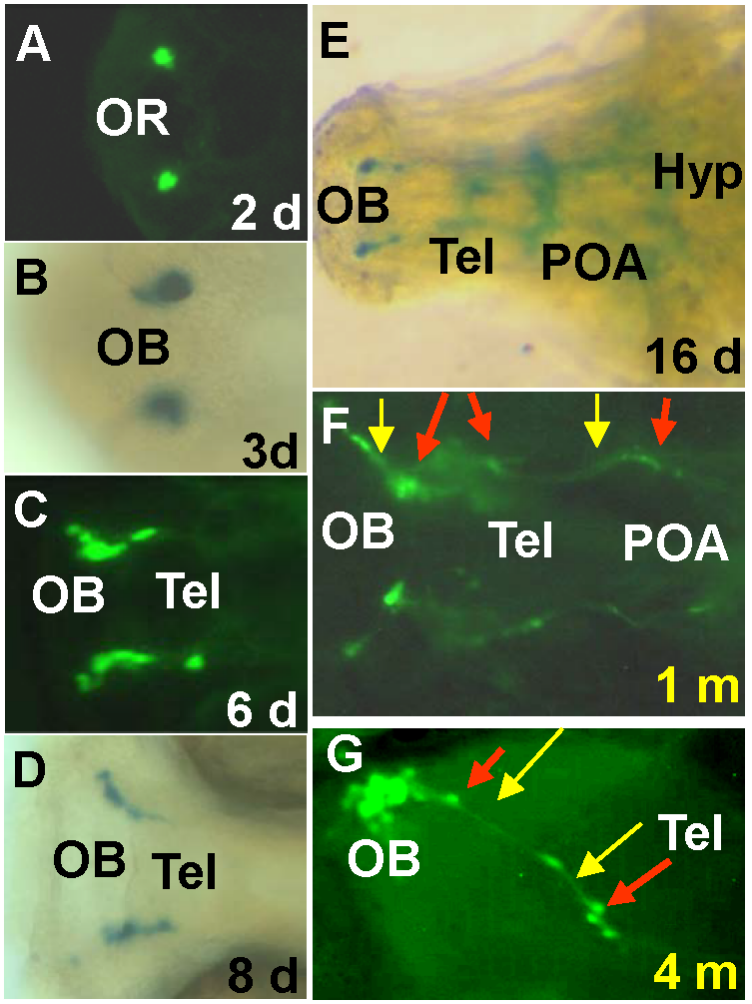
Detection of GFP- and LacZ-expressing cells in transgenic fish. **A**, GFP expression at the olfactory region (OR). **B–D**, GFP or LacZ cells in the olfactory bulb (OB) and telencephalon (Tel) at 3–8 d (days). **E**, lacZ cells and axons in the brain at 16 dpf. Hyp, hypothalamus. **F–G**, At 1 month (m) and 4 months, fluorescent axonal extensions are at OB, Tel and POA (preoptic area). Red arrows indicate cell bodies, yellow arrows indicate axons. A, C, F, G, GFP cells; B, D, E, LacZ-expressing cells. The anterior is towards the left in all panels. A, B, F, G, ventral view; C-E, dorsal view.

We have generated transgenic fish expressing GFP-LacZ under the control of the *gnrh3* promoter. Antibody staining detected GFP in the olfactory bulb of the transgenic fish, and this GFP expression co-localized with *gnrh3* transcripts both at 3 dpf and 4 dpf (Fig. 1). GFP expression started early at around 1 dpf, and was evident at 2 days postfertilization (dpf) at the olfactory region (Fig. 2A) and at the olfactory bulb at 3 dpf (Fig. 2B). The expression then extended towards telencephalon (Fig. 2C, D). The GFP/LacZ-expressing neurons were present in the telencephalon and preoptic area at 16 dpf and becoming stronger at 4 months of age (Fig. 2E–G). This GFP expression pattern is similar to those found in other *gnrh3*-GFP transgenic zebrafish [31], [32].

### Regulation of gnrh3 Neurons by *gnrh3*

**Figure 3:**
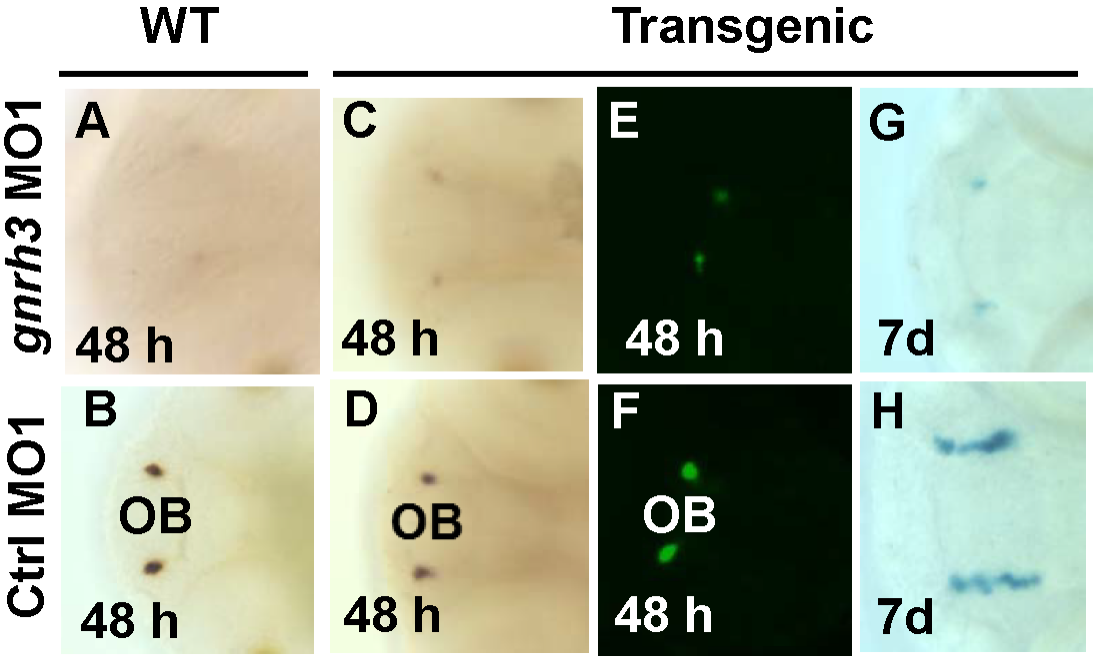
Depletion of *gnrh3* expression by morpholinos causes reduction of gnrh3 cells. A–D, *in situ* hybridization with the *gnrh3* probe. E–F, fluorescence detection of GFP cells. G–H, detection of LacZ-expressing cells. After injection of *gnrh3-*MO1, *gnrh3* and GFP expression was reduced at 48 hpf (A, C and E) and LacZ cells in the forebrain was reduced at 7 dpf (G). B, D, F and H, Gnrh3 cells neurons after the injection of control sense MO1 (Ctrl-MO1).

To understand the role of *gnrh3* in these gnrh3 neurons, we knocked down *gnrh3* expression in zebrafish with antisense morpholinos (MOs). This led to a decrease of gnrh3 cells at 48 hpf as detected by *in situ* hybridization (Fig. 3A–D). The expression of GFP fluorescence in transgenic fish that express *gnrh3:GFP-LacZ* was also decreased (Fig. 3E, F). At day 7, LacZ-expressing *gnrh3* cells were detected in control-MO1-injected larvae (Fig. 3H), but were missing after *gnrh3*-MO1 injection (Fig. 3G). In addition to *gnrh3*-MO1, a different morpholino, *gnrh3-*MO2, was also injected into zebrafish embryos, and 60% of the embryos had decreased gnrh3-expressing cells at 2 dpf. Similarly, *gnrh3-*MO3 resulted in a decrease of gnrh3-expressing cells in 30% of the embryos at 2 dpf. Therefore all three independent *gnrh3*-MO sequences led to reduced presence of gnrh3 cells.

**Figure 4:**
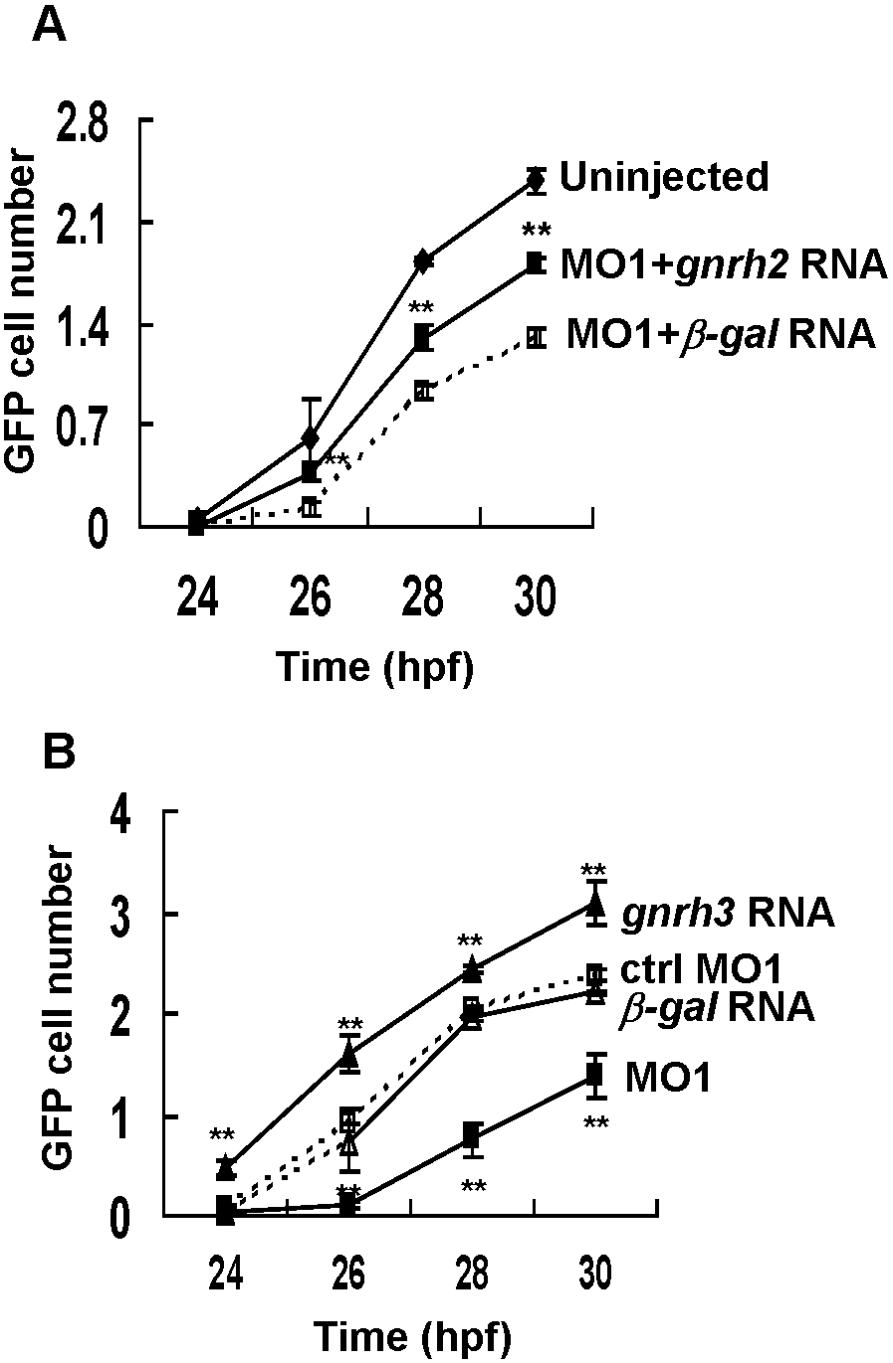
Gnrh3 controls the number of gnrh3 neurons. Gnrh3 neurons are detected as GFP expressing cells. A. The number of GFP cells at different time points after the injection of control MO, *gnrh3-*MO1, *gnrh3* RNA, *or β-gal* RNA. Depletion of *gnrh3* expression by *gnrh3-*MO1 (MO1) reduces the number of GFP cells, and over-expression of *gnrh3* increases GFP cell numbers. B, *gnrh2* mRNA rescues the *gnrh3*-MO1 morphants. Uninjected embryos and embryos injected with control (ctrl-MO1) and *β-gal* RNA are controls. *P<0.05. **P<0.01. Twenty embryos were counted for each data point, and the same injection experiments were repeated two to four times for each time point.

To rule out off-target effects of *gnrh3*-MOs, we added *gnrh* transcripts back to zebrafish embryos to see whether this can rescue the defect in *gnrh3* morphants. Because the *gnrh3*-MOs can bind *gnrh3* mRNA and destroy *gnrh3* mRNA, we rescued morphants with *gnrh2* mRNA because *gnrh2* mRNA is resistant to *gnrh3*-MO1 and gnrh2 can also bind to gnrh receptors. After injection of *gnrh2* mRNA into fertilized eggs, gnrh2 was mis-expressed in all parts of zebrafish embryos and partially rescued the defect of GFP cells caused by *gnrh3*-MO1 (Fig. 4A. Injection of a control *β-gal* mRNA together with *gnrh3-*MO1, however, still led to a reduction of GFP cells. This result indicated that gnrh has a role in the proliferation of gnrh3-expressing cells.

We also investigated the effect of *gnrh3* overexpression on the expansion of gnrh3 cells. After *gnrh3* mRNA microinjection, GFP cells appeared earlier and their cell numbers increased faster than those in the control fish injected with control *β-gal* RNA (Fig. 4B). These data indicated that *gnrh3* was important for the differentiation and proliferation of gnrh3-expressing neurons.

### Regulation of gnrh3 Neurons by the Hedgehog-PKA Pathway

**Figure 5:**
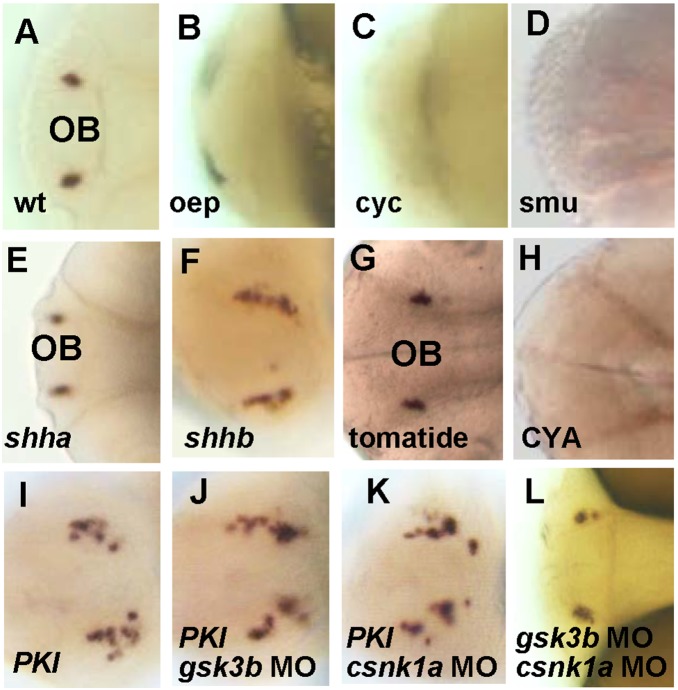
Hh-PKA pathway is required for the development of gnrh3 neurons. All embryos are at 2**A–D**, *gnrh3* neurons were detected by *in situ* hybridization with the *gnrh3* probe in the wildtype (wt), but not in *oep, cyc* and *smu* mutants. **E–F**. Ectopic GFP expression near the olfactory region in embryos injected with (F) *shhb*, but not (E) *shha* mRNA. **G–H**, *gnrh3* signals were normal in control (G) tomatide-treated embryos, but were lost in embryos treated with (H) Hh inhibitor cyclopamine (CYA, 100 µM) at 6–10 hpf. **I–K**, More ectopic *gnrh3*-expressing cells were detected after co-injection of *gsk3b*-MO or *cnsk1a*-MO with *PKI* mRNA. **L**, *Gsk3b* and *csnk1a* double MO treatment did not increase the number of ectopic *gnrh3*-expressing cells. The anterior is to the left in all panels.

We further investigated the developmental control of gnrh3 neurons by screening existing mutants for defective *gnrh3* expression. Expression of *gnrh3* was not detected in about a quarter of offspring from the crosses of *oep*, *cyc*, and *smu* heterozygous parents (Fig. 5A–D). The scoring of these mutants followed a Mandelian ratio (Table 1), indicating that these mutants affect something linked to the appearance of the gnrh3 cells. The *oep* and *cyc* mutants do not possess ventral forebrain and thus lose *shh* expression [24], [25], while *smu* is defective in Hh signaling [26]. We therefore examined the participation of Hh signaling in more detail.

**Table 1:** Aberrant gnrh3 neuron numbers in embryos with perturbed Hh signaling pathways.

Mutants or treatment	% embryos with decreased *gnrh3* expression after incross	Hh manipulation	% embryos with ectopic GFP expression	% embryos with ectopic *gnrh3* expression
oep	21% (n = 95)	*PKI*	9% (n = 34)	11% (n = 45)
oep+*PKI*	2% (n = 108)	*PKI* + *gsk3b*-MO	29% (n = 38)	29% (n = 34)
cyc	24% (n = 127)	*PKI* + *csnk1a*-MO	26% (n = 31)	31% (n = 35)
cyc+*PKI*	1% (n = 126)	*gsk3b*-MO	0% (n = 30)	0%
smu	27% (n = 59)	*csnk1a*-MO	0% (n = 30)	0%
		*gsk3b*-MO + *csnk1a*-MO	0% (n = 30)	0%

Embryos from heterozygote mating were examined for *gnrh3* expression and the numbers of embryos were scored. Alternatively embryos from wildtype parents were injected with morpholinos or mRNAs before scoring ectopic GFP and *gnrh3* expression at 2 dpf.

When *shha* (*sonic hedgehog a*, *syu*) mRNA was injected into zebrafish embryos, the pattern of *gnrh3* was the same as that found in wildtype embryos (Fig. 5E). However, when *shhb* (*sonic hedgehog b*) was misexpressed in zebrafish embryos, increased numbers of ectopic GFP cells were detected (Fig. 5F). Conversely, when fish embryos were incubated with Hh pathway blocker cyclopamine (CYA) from 6–10 hpf, the number of gnrh3 neurons was decreased, while incubation with control chemical tomatide had no effect (Fig. 5G–H, Table 2). These gain- and loss-of function studies indicate the participation of shhb signaling in the development of gnrh3 neurons.

**Table 2:** Aberrant numbers of gnrh3 neurons in embryos treated with different chemicals.

Treatment	Treatment Time	% *gnrh3* ^+^ embryos	% embryos with ectopic *gnrh3* expression
Cyclopamine (100 µM)	6–8 hpf	45% (n = 42)	0%
	6–10 hpf	0% (n = 126)	0%
	6–24 hpf	0% (n = 49)	0%
Cyclopamine (50 µM)	6–8 hpf	93% (n = 54)	0%
	6–10 hpf	13% (n = 32)	0%
	6–24 hpf	0% (n = 28)	0%
Tomatidine (100 µM)	6–10 hpf	100% (n = 84)	0%
	6–24 hpf	100% (n = 31)	0%
LiCl (200 µM)	6–7 hpf	50% (n = 37)	0%
	6–8 hpf	0% (n = 51)	0%
	7–8 hpf	100% (n = 29)	0%
	6–10 hpf	0% (n = 38)	0%
	7–9 hpf	62% (n = 13)	0%
	8–10 hpf	100% (n = 43)	0%
	8–12 hpf	100% (n = 42)	50%
	8–24 hpf	100% (n = 28)	100%
	10–24 hpf	100% (n = 41)	0%

Embryos were treated with drugs for different time periods, and the numbers of embryos that expressed *gnrh3* were scored.

To dissect the involvement of Hh signaling pathway in gnrh3 cell development, we further examined the participation of kinases that antagonize the Hh pathway. In Drosophila, the Hh signaling is blocked by three kinases, PKA, CK1 and GSK3b [33]. In zebrafish the Hh signaling is also antagonized by the PKA pathway, which can be inhibited by a dominant-negative regulatory subunit of *protein kinase A* (PKI) [28]. Blocking PKA function by *PKI* caused ectopic *gnrh3* expression in about 10% embryos (Fig. 5I and statistics at Table 1). Co-injection of *PKI* mRNA and *gsk3b*-MO also increased the number of cells that express *gnrh3* ectopically (Fig. 5J). Similarly co-injection of MO against *csnk1a* (zebrafish *CK1* orthologue) with *PKI* mRNA also increased ectopic *gnrh3* expression (Fig. 5K), and the population of embryos with increased *gnrh3* expression increased to about 30% (Table 1). Injection of *gsk3b*-MO and *cnsk1a*-MO, alone or together, however, did not cause ectopic *gnrh3* expression (Fig. 5L, Table 1). Blocking GSK3b activity by LiCl at 6–8 hpf or 6–10 hpf completely blocked *gnrh3* expression, but LiCl treatment at 8–12 hpf or 8–24 hpf caused ectopic *gnrh3* expression (Table 2), implying that GSK3b affects the differentiation of *gnrh3* neurons differently at different developmental stages. These data suggest the PKA, CK1 and GSK3b pathways regulate the development of gnrh3 neuronal progenitors.

**Figure 6:**
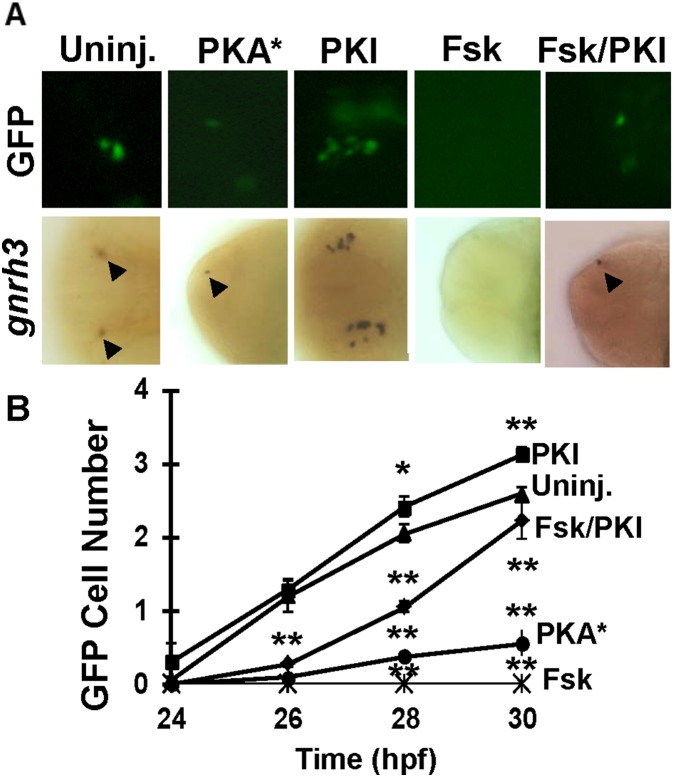
PKA pathway antagonizes the development of gnrh3 neurons at 30 hpf. **A**, GFP- (top) or *gnrh3*-expressing (bottom) cells in embryos injected with mRNA for constitutively active subunit of *PKA* (PKA*), *PKI*, or treated with forskolin (Fsk) or *PKI* + forskolin. The anterior is to the left in all panels. **B**, Quantitation of the numbers of GFP-expressing neurons. Twenty embryos were counted for each data point. *P<0.05. **P<0.01.

To further examine the effect of PKA signaling on *gnrh3* expression, we increased PKA activity by injected into fish embryos mRNA for the constitutively active catalytic subunit of PKA (PKA*). This led to a decrease of GFP- and LacZ-expressing cells at 30 hpf (Fig. 6). *PKI* mRNA (100 pg /embryo) injection, on the contrary, increased the number of ectopic GFP- and LacZ-expressing cells. High concentrations of PKA activator forskolin (200 µM) decreased the number of GFP/LacZ/gnrh3 cells (Fig. 6). *PKI* mRNA microinjection also partially rescued *gnrh3* cells in embryos treated with forskolin treatment (Fig. 6) or in *oep* and *cyc* mutant (Fig. S3 in File S1).

### Effect of Fgf Pathway in the Development of gnrh3 Neurons

**Figure 7:**
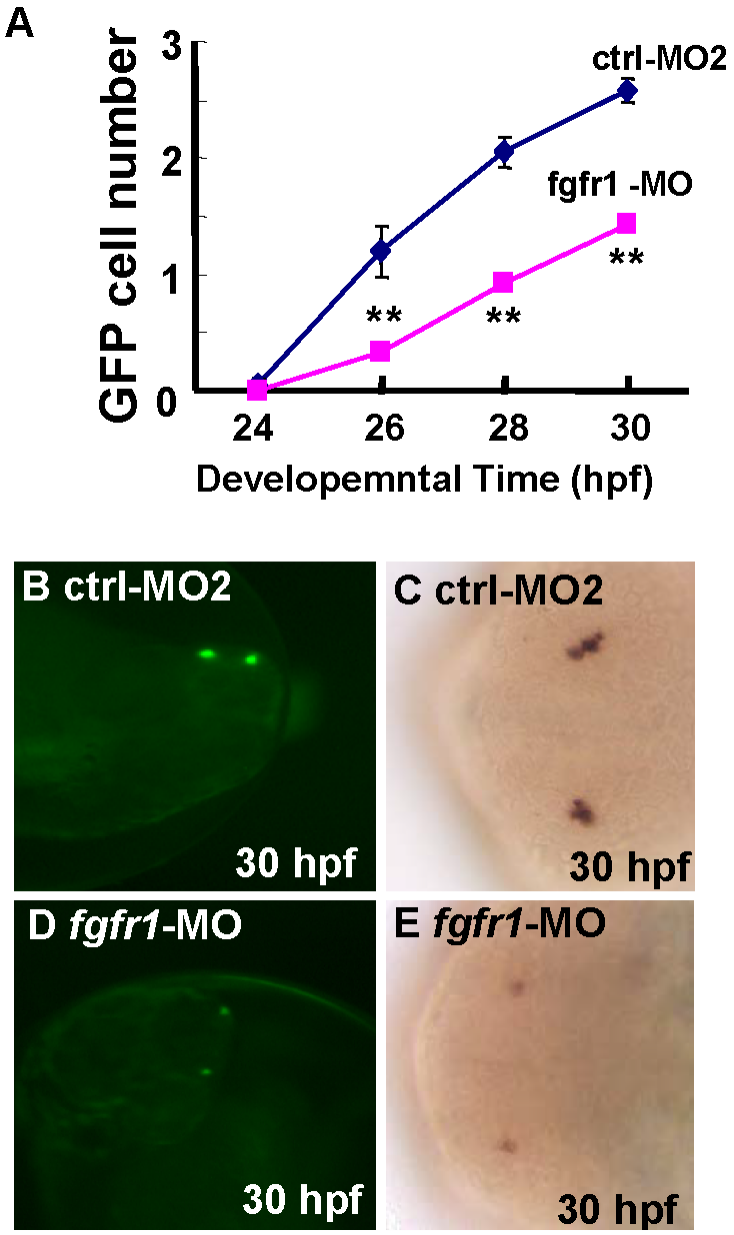
Requirement of Fgf pathway in the development of gnrh3 neurons. **A**, Decreased GFP cell numbers after injection of *fgfr1*-MO as compared with embryos injected with control MO2 (ctrl-MO2). **B–E**, Decreased *gnrh3*-expressing cells as detected by (B, D) GFP- or (C, E) *in situ* hybridization. The anterior is to the left in all panels. Twenty embryos were counted for each data point. **P<0.01.

We also tested Fgf pathway in gnrh3 neuron development by *fgfr1*-MO microinjection. The *fgfr1*-MO treatment caused a decrease in the number of gnrh3 neurons, whereas control-MO2 had no effect (Fig. 7). This result suggests that the Fgf pathway may be involved in gnrh3 neuron development.

## Discussion

In this paper, we generated GFP-LacZ transgenic fish that express GFP and LacZ in *gnrh3*-expressing cells. This fish line was used to analyze the development of gnrh3 neurons. We showed that the differentiation and proliferation of these cells were controlled by gnrh3 itself and by the Hh-PKA signaling pathway.

We showed the effect of the Hedgehog-PKA pathway in the development of gnrh3-expressing cells by inhibitor treatment, analysis of fish mutants, morpholino knockdown, and mRNA overexpression experiments. Hedgehog signaling regulates the patterning of craniofacial neural crest cells [34], which will become gnrh3 progenitors located adjacent to the olfactory placode [8]. The Hedgehog signaling may regulate the patterning and the spatial distribution of GnRH3 neurons by influencing neural crest cell migration and/or differentiation. The effect of hedgehog signaling on gnrh cell fate, however, is not that simple. Zebrafish has two shh genes, *shha* and *shhb.* While *shhb* mRNA misexpression led to ectopic gnrh3 progenitor cells, *shha* misexpression had no such effect. This data indicates that the functions of these two genes are not identical, and *shhb* seems to be more instructive than *shha* in directing gnrh cell expansion. Detailed functions of the Hh pathway in zebrafish still need to be examined in the future.

Multiples signaling molecules appear to be involved in the differentiation of ghrn3 neurons. We found blocking of FGF pathway led to a reduction of gnrh3 cells. Furthermore, mis-expression of constitutively active PKA* prevented the expansion of gnrh3-expressing cells from their progenitors. Blocking PKA by *PKI* or *PKI* together with *gsk3b*/*cnsk1a* morpholinos increased ectopic expression of gnrh3 neurons. GSK3b inhibitor, LiCl, abolished or caused ectopic *gnrh3* expression in the zebrafish at different developmental states (Table 2). GSK3b and CK1 also participate in the Wnt pathway [35]; it is possible that WNT pathway may also regulate the development of *gnrh3* neurons.

Several signaling molecules including BMP, FGF, Wnt, and Hh are expressed in the neural plate close to the preplacodal field or in primordial sensory organs close to the cranial placode [36]. Individually or in combination, these molecules are candidates that may induce the formation of cranial preplacode, as well as of individual cranial placodes [37]–[39]. Detailed functions of Hh or other signaling pathways in gnrh3 neurons still need to be examined in the future.

In this report we show that *gnrh3* is important for the development of gnrh3 neurons, as depletion of *gnrh3* causes reduction of gnrh3 cell numbers in zebrafish. Gnrh3 is important for the migration, fiber development and pathfinding of gnrh3 neurons [15]. It is also important for the maintenance of gnrh3 neurons [40]. We have blocked gnrh3 expression by morpholinos since fertilization and shown the importance of gnrh3 in the expansion of gnrh3 neurons at 24–30 hfp. Thus, gnrh3 is important for both the proliferation and the maintenance of gnrh3 neurons. GnRH secreted by olfactory neuroblast cell line FNC-B4 also acts in an autocrine manner to promote the differentiation and migration of GnRH-secreting neurons [41]. These data indicate the conserved autocrine role of GnRH for the development of GnRH neuron system.

## Supporting Information

File S1 **Contains Figures S1, S2, and S3.** Figure S1. Similar patterns between LacZ staining and GFP signals at different stages. Figure S2. Structure of the *gnrh3* mRNA, its gene, and the design of plasmid to generate transgenic fish expressing *GFP-LacZ* under the control of the *gnrh3* promoter. Figure S3. PKI rescue the gnrh3 neurons in *oep* or *cyc* mutants at 30 hpf. A, Examination of gnrh3 cell numbers in *PKI-*injected *oep* mutant. B, Examination of gnrh3 cell numbers in *PKI-*injected *cyc* mutant.
(DOCX)
