# Supplementary material for: Hedgehog-PKA Signaling and gnrh3 Regulate the Development of Zebrafish gnrh3 Neurons
Source: PLoS One. 2014 May 30;9(5):e95545. doi: 10.1371/journal.pone.0095545 (PMC4039432; doi:10.1371/journal.pone.0095545)
Supplement: File S1 — Contains Figures S1, S2, and S3. Figure S1. Similar patterns between LacZ staining and GFP signals at different stages. Figure S2. Structure of the gnrh3 mRNA, its gene, and the design of plasmid to generate transgenic fish expressing GFP-LacZ under the control of the gnrh3 promoter. Figure S3. PKI rescue the gnrh3 neurons in oep or cyc mutants at 30 hpf. A, Examination of gnrh3 cell numbers in PKI-injected oep mutant. B, Examination of gnrh3 cell numbers in PKI-injected cyc mutant. (DOCX) [file pone.0095545.s001.docx]

**Supplementary Figures**

**Fig. S1. Similar patterns between LacZ staining and GFP signals at different stages.** Lac Z staining are shown in zebrafish with PTU treatment. The GFP signals are expressed in zebrafish without PTU treatment. LacZ staining and GFP signals were observed at 8 dpf (upper two panels) and 21 dpf (lower two panels).


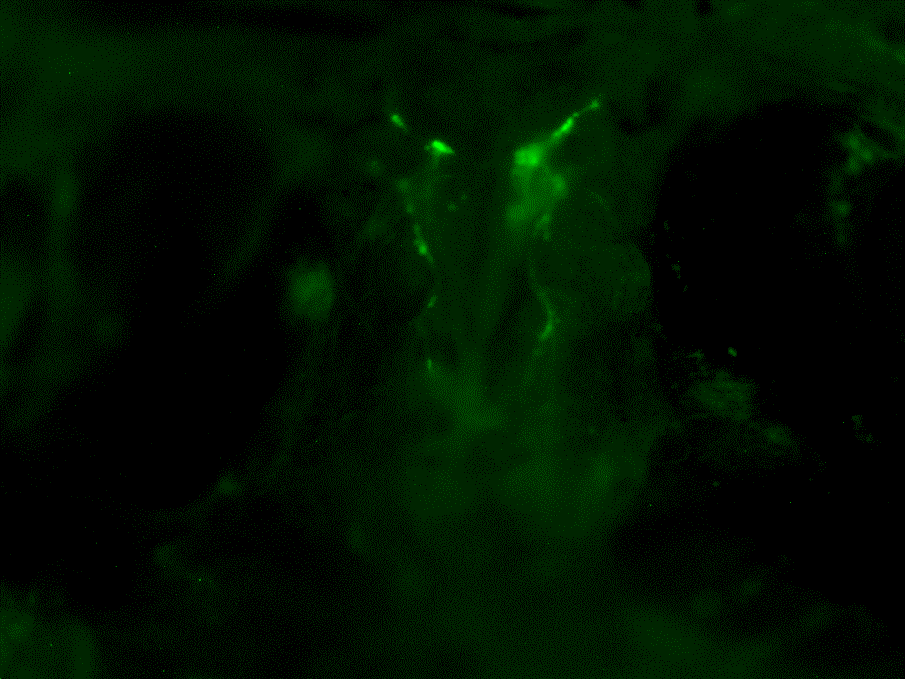

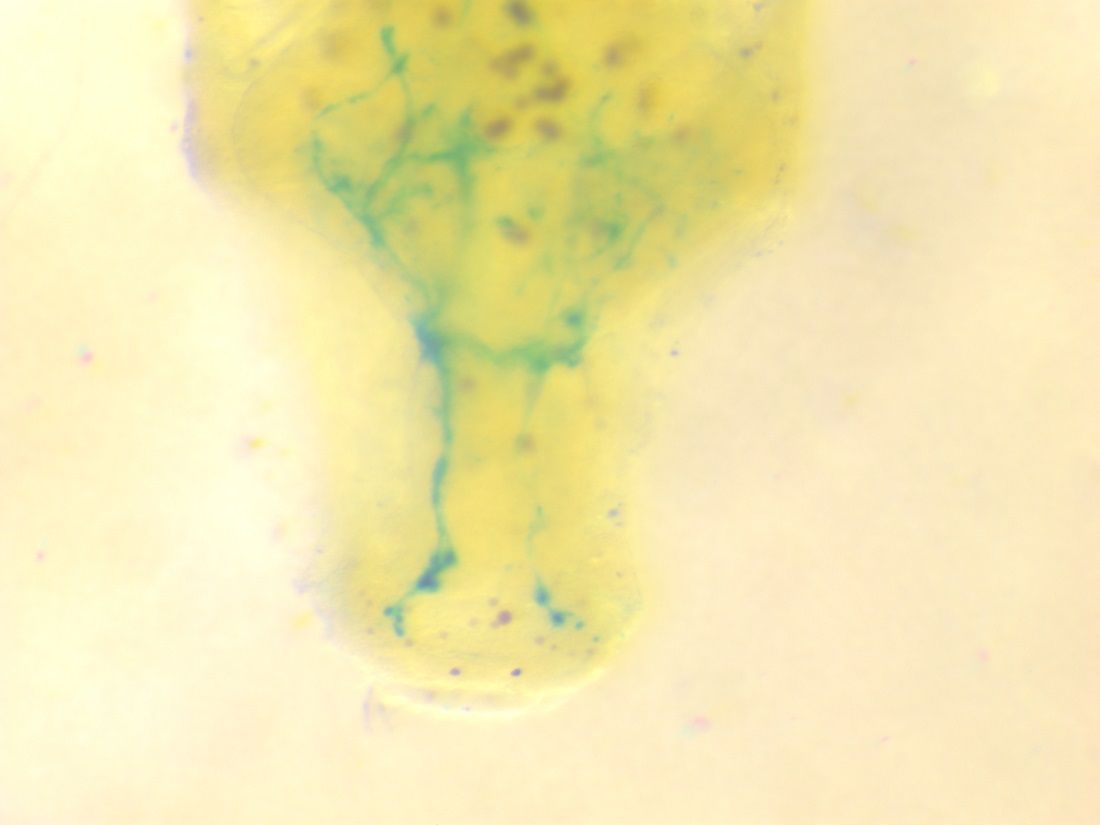

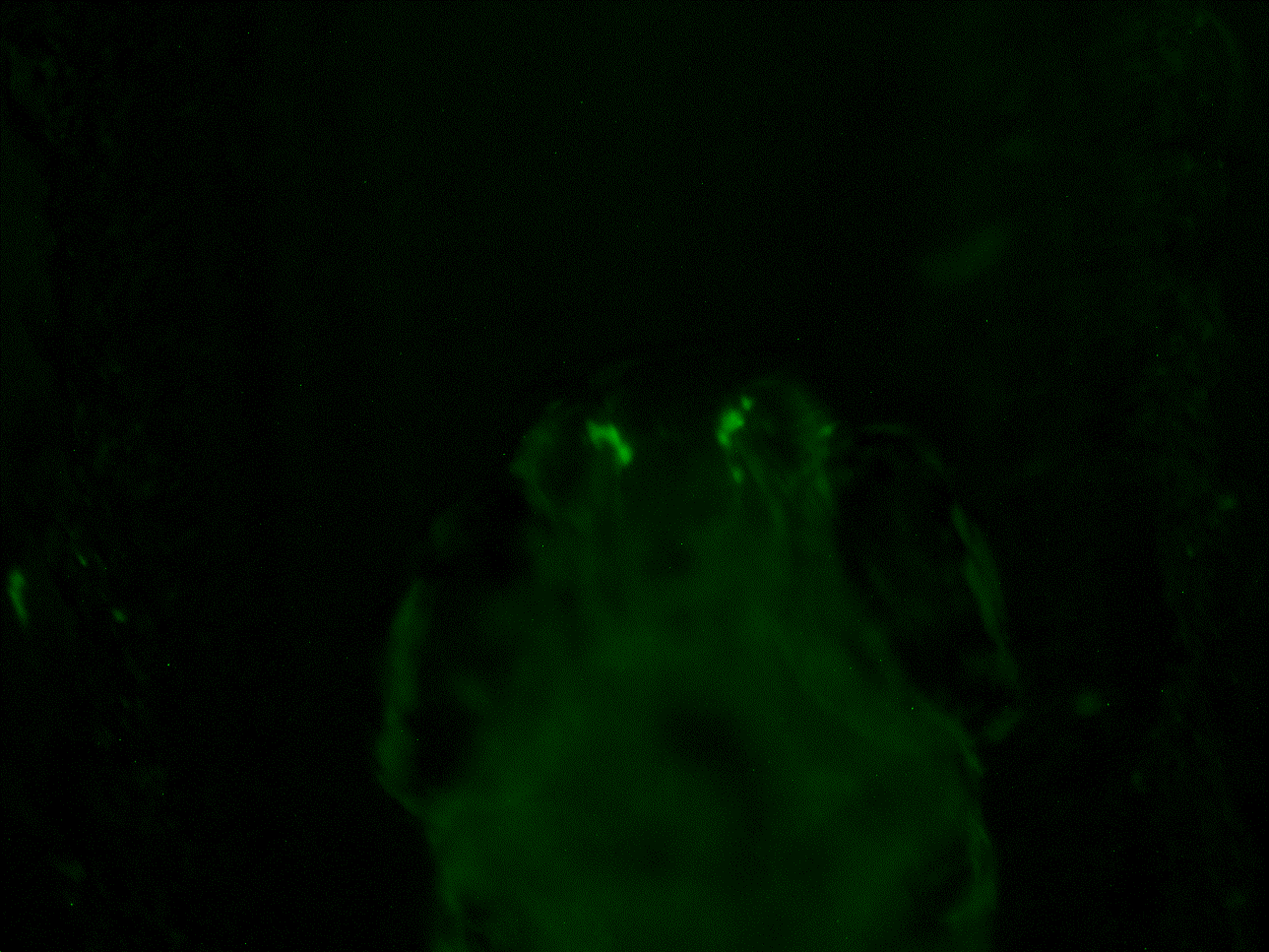

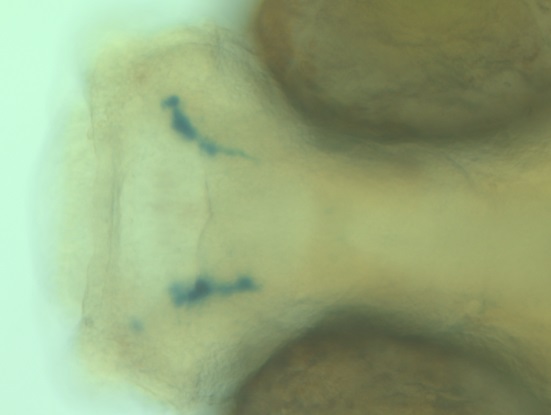


**GFP/No PTU**

**21 dpf**

**8 dpf**

**LacZ/PTU**

**Fig. S2. Structure of the *gnrh3* mRNA, its gene, and the design of plasmid to generate transgenic fish expressing *GFP-LacZ* under the control of the *gnrh3* promoter.** The gnrh3 preprohormone and cDNA are 94 a.a. and 430 bp, respectively. The *gnrh3* gene consists of four exons (20, 145, 75 and 193 bp each) and three introns (1129, 1509 and 94 bp each). The 5’-*gnrh3* fragment (2.7 kb) and 3’-*gnrh3* fragment (2.6 kb) were cloned into the *pChi-GZK* plasmid for the generation of transgenic fish. Sequences for morpholino MO1 to MO3 are indicated as lines with their directions shown by arrows.


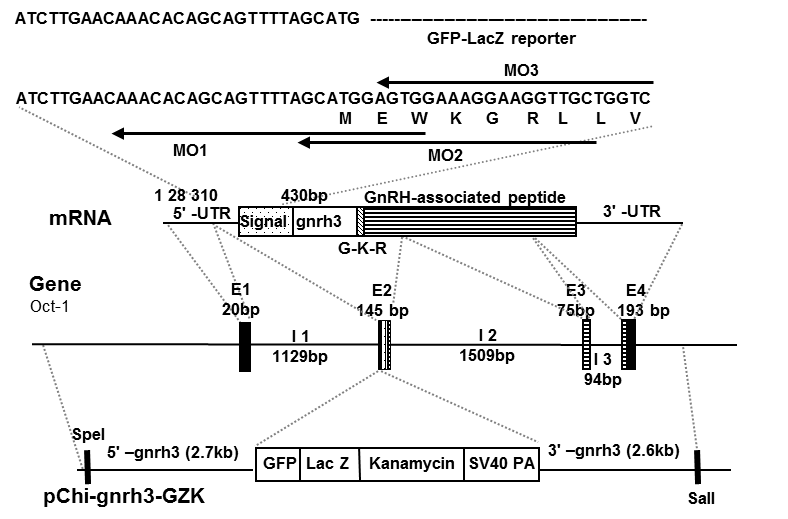


**Fig. S3. PKI rescue the gnrh3 neurons in *oep* or *cyc* mutants at 30 hpf.** A, Examination of gnrh3 cell numbers in *PKI-*injected *oep* mutant. B, Examination of gnrh3 cell numbers in *PKI-*injected *cyc* mutant.


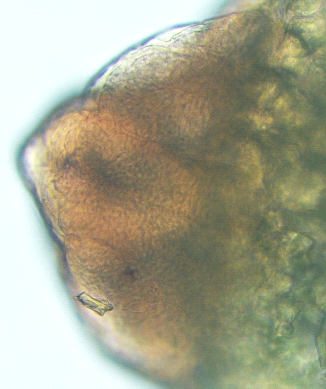

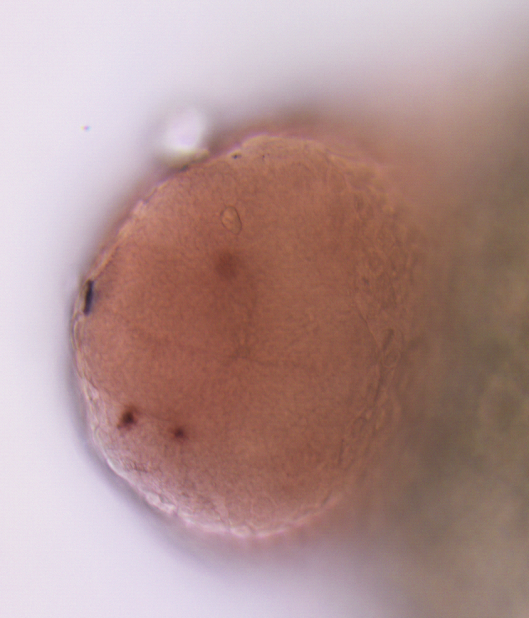


***oep***

**+*PKI*/30hpf**

***cyc***

**+*PKI*/30hpf**

**99%, n =126**

**98%, n =**

**108**
